# Impact of Voluntary Alcohol Consumption on Corticostriatal Plasticity in Rats

**DOI:** 10.1111/ejn.70602

**Published:** 2026-07-02

**Authors:** Johanna A. S. Smeets, Geert M. J. Ramakers, Frank J. Meye, Anne‐Marie Baars, Roger A. H. Adan, Louk J. M. J. Vanderschuren, Heidi M. B. Lesscher

**Affiliations:** ^1^ Department of Population Health Sciences, Unit of Animals in Science and Society, Faculty of Veterinary Medicine Utrecht University Utrecht the Netherlands; ^2^ Department of Translational Neuroscience, University Medical Center Utrecht Brain Center Utrecht University Utrecht the Netherlands

**Keywords:** alcohol, brain slices, optogenetics, patch‐clamp electrophysiology, plasticity, rats

## Abstract

Alcohol use disorder (AUD) is a chronic relapsing disorder that is characterized by loss of control over alcohol consumption. Loss of control over alcohol use has been proposed to be mediated by a combination of habitual substance use, caused by functional changes in the dorsolateral striatum (DLS), and breakdown of cognitive control over alcohol use, subserved by cortical areas. We have previously shown that a subgroup of Lister Hooded rats develops loss of control over alcohol seeking after voluntary intermittent‐every‐other‐day consumption of alcohol. The aim of this study was therefore to determine the effects of long‐term voluntary alcohol consumption on sensorimotor cortical inputs to the DLS in subgroups of rats that had consumed low versus high amounts of alcohol. To that end, optogenetics and patch‐clamp electrophysiology were combined to investigate functional changes in cortical projections to the DLS after 8 weeks of voluntary alcohol consumption. We observed lower AMPA/NMDA ratios in alcohol‐consuming rats, indicative of long‐term depression of this projection upon exposure to alcohol. We observed a reduced coefficient of variance and, selectively in high alcohol drinking rats, an increase in paired‐pulse facilitation. This indicates that voluntary alcohol consumption induces both post‐ and presynaptic changes in the sensorimotor cortical inputs to the DLS. These findings show that alcohol consumption affects corticostriatal plasticity, which might contribute to the development of AUD.

## Graphical abstract

After 8 weeks voluntary alcohol consumption, AMPA/NMDA ratios were reduced in medium spiny neurons in the dorsolateral striatum, upon optogenetic stimulation of sensorimotor cortical inputs. Moreover, coefficient of variance was reduced and paired‐pulse facilitation was enhanced, selectively in high alcohol drinking rats. These findings suggest that voluntary alcohol consumption induces presynaptic changes in the sensorimotor cortical inputs to the DLS, underlining the impact of alcohol consumption on corticostriatal plasticity.

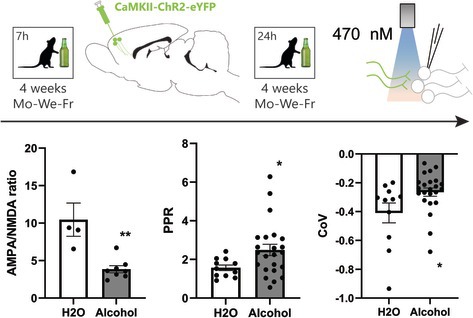

**Abbreviations**
AAV: adeno‐associated virus
ACSF: artificial cerebrospinal fluid
AP: anterior–posterior
AUD: alcohol use disorder
BDNF: brain‐derived neurotrophic factor
CNQX: cyanquixaline (6‐cyano‐7‐nitroquinoxaline‐2,3‐dione)
CoV: coefficient of variance
DLS: dorsolateral striatum
DV: dorsal‐ventral
EPSC: excitatory postsynaptic current
GFP: green fluorescent protein
I.P: intraperitoneal
LTD: long‐term depression
ML: medial‐lateral
PFC: prefrontal cortex
PPR: paired‐pulse ratio

## 1 Introduction

Alcohol use disorder (AUD) is a chronic relapsing disorder that is characterized by a lack of control over alcohol consumption. AUD gives rise to medical, socioeconomic, and legal challenges, thereby contributing substantially to the global disease burden (Connor et al. 2016; Rehm et al. 2009). The pathology of AUD is diverse, and its diagnosis relies on various behavioral criteria such as persistent alcohol use despite negative consequences, and the presence of craving (American Psychiatric Association 2013). Despite the high prevalence and costs to society, treatment options for AUD are limited in number and efficacy (O'Brien 2008; van den Brink 2012). Moreover, the available treatments are not directed at restoring control over alcohol use. Better understanding of the pathophysiology of AUD, including its underlying neurobehavioral structure, may contribute to the development of more effective prevention and treatment strategies for this disorder.

Loss of control over substance use has been proposed to be mediated by a combination of habitual substance use, caused by functional changes in the dorsolateral striatum (DLS), and breakdown of cognitive control over substance use, subserved by cortical areas (Barker et al. 2015; Bechara 2005; Everitt and Robbins 2005, 2013, 2015; Garavan and Stout 2005; Goldstein and Volkow 2011; Jentsch and Taylor 1999; Johnson et al. 2020; Kalivas and Volkow 2005; Koob and Volkow 2010; Perry and Carroll 2008; Pierce and Vanderschuren 2010; Rangel‐Barajas et al. 2021; Smith and Laiks 2018). The DLS has been shown to drive (excessive) alcohol drinking and operant ethanol self‐administration in rodents (e.g., Bahi and Dreyer 2013; Bauer et al. 2021; Darcq et al. 2016; Giuliano et al. 2019; Jeanblanc et al. 2015), although the DLS may no longer drive these behaviors after an extended history of alcohol consumption (Bauer et al. 2022). Multiple animal studies indicate that altered function of the DLS contributes to loss of control over substance seeking and taking (Belin and Everitt 2008; Corbit et al. 2012; Jonkman et al. 2012; Murray et al. 2012; Vanderschuren et al. 2005; Zapata et al. 2010). Habitual alcohol seeking has been shown to be mediated by a shift in response control from the dorsomedial to the dorsolateral striatum (Corbit et al. 2012; Lipton et al. 2019). The dorsolateral, or sensorimotor, striatum has been shown to mediate habit formation (O'Hare et al. 2016; Yin and Knowlton 2006). For example, habitual alcohol seeking relies on both AMPA and dopamine D2 receptor signaling in the DLS (Corbit et al. 2014; Giuliano et al. 2019). Furthermore, ablation of striatal fast‐spiking interneurons in the DLS was shown to reduce compulsive alcohol consumption (Patton et al. 2021), further emphasizing the involvement of the DLS in loss of control over alcohol use. Importantly, chronic exposure to alcohol has been demonstrated to cause morphological and functional neuroadaptations in the DLS. This is evident from greater dendritic complexity, greater dendritic length and attenuated or even abolished long‐term depression (LTD) upon forced exposure to alcohol via drinking water or vapor (Cui et al. 2011; DePoy et al. 2013; DePoy et al. 2015; Xia et al. 2006). The DLS is largely innervated by the sensorimotor cortex (S1, S2, M1, and M2), and an essential function of the DLS is to convert sensorimotor cortical input into activity of output neurons that feed into the direct and indirect pathways of the basal ganglia (Alloway et al. 2017; Brown et al. 1998; Voorn et al. 2004). The sensorimotor circuit (S1, M1, and M2) in and of itself has been shown to be engaged during training and habit formation (Michiels et al. 2026), and the sensorimotor‐DLS circuit has been implicated in habitual substance use (Gremel and Lovinger 2017; Sitzia and Lovinger 2023). However, there is limited empirical data regarding the involvement of the sensorimotor‐DLS projection in habitual substance seeking.

The aim of this study was therefore to determine the effects of long‐term voluntary alcohol consumption on glutamatergic corticostriatal transmission and plasticity in subgroups of rats that consumed low versus high amounts of alcohol. To that end, we investigated functional changes in projections from the sensorimotor cortex (M1 and M2) to the dorsolateral striatum after 8 weeks of alcohol consumption. We have previously shown individual variation in alcohol consumption that predicts the degree of loss of control over alcohol seeking after 8 consecutive weeks of voluntary intermittent‐every‐other‐day alcohol consumption (Lesscher et al. 2021; Spoelder et al. 2017; Spoelder et al. 2015). We hypothesized that glutamatergic corticostriatal plasticity would be augmented particularly in high alcohol consuming rats.

## 2 Materials and Methods

### 2.1 Subjects

Male Lister‐hooded rats (Charles River, Germany) weighing 250–300 g at the start of the experiment were individually housed in Macrolon type III sawdust bedded cages (42.5 × 26.6 × 18.5 cm). The animals were housed in a humidity‐ and temperature‐ controlled environment (*21 ± 2°C and 50%–70% humidity*) under a 12 h: 12 h reversed day/night cycle (lights off at 7 AM) and had ad libitum access to water and chow (Rat and Mouse Breeder and Grower Expanded‐CRM(E), Special Diet Service, United Kingdom). The rats were acclimatized to the housing conditions for at least 8 days prior to behavioral testing. The animals were handled and weighed at least once per week. All experiments were approved by the Animal Ethics Committee of Utrecht University and conducted in agreement with Dutch laws (Wet op de Dierproeven, 1996, revised 2014) and European regulations (Guideline 86/609/EEC; Directive 2010/63/EU).

### 2.2 Experimental Design

**FIGURE 1:**
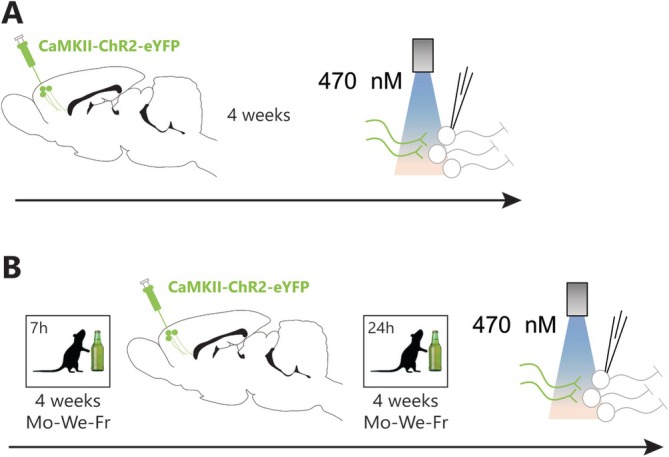
Procedural outline. (A) Schematic representation of the first experiment where channelrhodopsin‐expressing AAV particles were infused into the sensorimotor cortex. Four weeks after surgery, the rats were euthanized for in vitro electrophysiological recordings from medium spiny neurons in the DLS upon optical stimulation of the sensorimotor cortex projections to the DLS using 470‐nm light pulses. (B) Outline for the second experiment where rats were allowed to consume alcohol in 7‐h sessions on Mondays, Wednesdays, and Fridays for four consecutive weeks. Thereafter, the extreme low and high animals were selected and received a local infusion of the channelrhodopsin‐expressing AAV particles in the sensorimotor cortex. Thereafter alcohol consumption was resumed for another 4 weeks in 24‐h sessions on Mondays, Wednesdays, and Fridays. Thereafter, the rats were euthanized for in vitro electrophysiological recordings from medium spiny neurons in the DLS upon optical stimulation of the sensorimotor cortex projections to the DLS using 470‐nm light pulses.

In this study, optogenetics and electrophysiology were combined in rats that consumed alcohol voluntarily for 8 weeks, to determine the impact of alcohol intake on glutamatergic corticostriatal transmission and plasticity. The outline of the experiments is summarized in Figure 1 and a detailed description of the experimental procedures will be provided in the subsequent sections.

**FIGURE 2:**
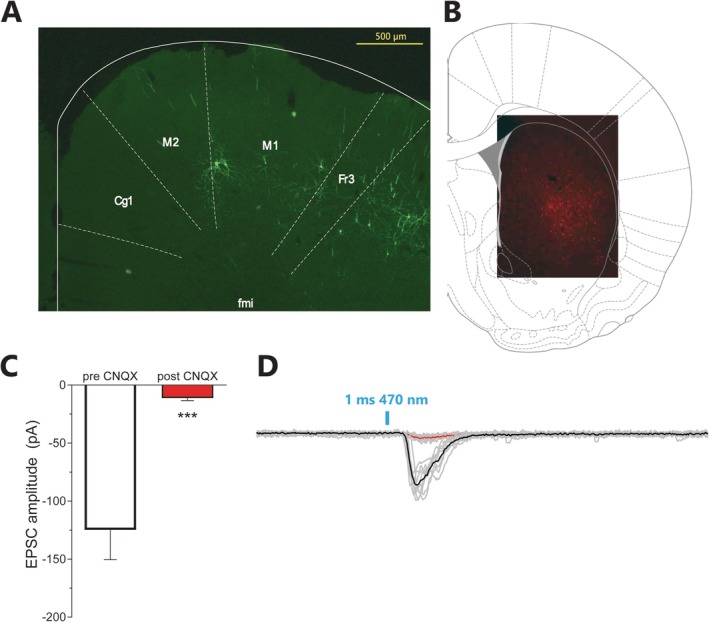
Sensorimotor input to the DLS. (A) GFP‐tagged retrograde labelled rabies virus in the sensorimotor cortex after local infusion in the DLS, confirming innervation of the DLS by the sensorimotor cortex. (B) Shows mCherry expression in the DLS as an example of the rabies infusion site, because AAV‐hSyn‐mCherry was co‐infused with Rabies‐SadDG‐GFP. (C) Average + SEM EPSC amplitudes and (D) traces of recordings for medium spiny neurons in the DLS upon optical stimulation using 470‐nm light pulses to active the sensorimotor cortical projections to the DLS in the absence (black) or presence (red) of the AMPA/kainate receptor antagonist CNQX (10 μM). *n* = 2 cells. Data expressed as mean ± SEM. Asterisk (*) denotes significance from pre CNQX at a *p* < 0.001 level.

Briefly, in the first experiment the anatomical projections to the DLS were visualized by local infusion of a mixture of a GFP‐tagged retrograde travelling rabies virus and AAV‐mCherry into the DLS (*n* = 4, Figure 2A,B). Subsequently, channelrhodopsin‐expressing AAV was infused in the sensorimotor cortex. Four weeks after surgery, the rats were euthanized for in vitro electrophysiological recordings. Medium spiny neurons in the DLS were recorded and afferent fibers from the sensorimotor cortex were stimulated using 470‐nm light pulses (*n* = 2, see below for detailed description) and light‐evoked synaptic responses were recorded. Bath application of CNQX was used to determine the glutamatergic nature of the light‐evoked EPSC's (Figure 2C,D).

In the second experiment, a total of 64 rats (divided over 2 batches of 40 and 24 rats, respectively) were allowed to consume alcohol in the homecage according to an intermittent‐every‐day schedule (Figure 1B). After the first 4 weeks of 7‐h drinking sessions, the extreme low and high alcohol drinking rats were selected based on a quartile split; the medium alcohol drinking rats were excluded from further testing. An additional 20 water control rats were included. These control animals consumed only water but were otherwise treated similarly to their alcohol consuming counterparts. The animals were infused with the channelrhodopsin‐expressing AAV particles in the sensorimotor cortex, and after 1 week for recovery the rats resumed with 24‐h intermittent alcohol consumption (or water only for the controls) sessions for another 4 consecutive weeks. Thereafter, the rats were euthanized for electrophysiological recordings (see below).

### 2.3 Voluntary Home Cage Alcohol Consumption

The rats were therefore given intermittent access to alcohol (IAA) and water in a two‐month two‐bottle choice setup in the home cage (Figure 1B). During each alcohol drinking session, the rats were presented with two bottles containing an alcohol solution (20% ethanol, v/v in tap water) (Klinipath, the Netherlands) and water. Drinking sessions occurred 3 days a week for 7 h/day during the first month of the paradigm (starting at 9 AM, i.e., 2 h after the onset of the dark phase) and were extended to 24 h/day during the second month of the paradigm, after the stereotaxic surgeries. Alcohol was freshly diluted with tap water to a final concentration of 20% (v/v) once per week. The position of the bottles was changed between drinking sessions to avoid a side‐preference. The bottles were weighed before and after each drinking session. Fluid intake was calculated by subtracting the bottle weights at the end of every drinking session by the starting weights. The rats in the water control group were treated like their alcohol drinking counterparts, except that they received two bottles of water instead.

Alcohol intake (ml) was calculated using the following equation: (Δ alcohol bottle weight in grams) / (0.8 + (0.2 × 0.789)) in which the density of ethanol (i.e., 0.789 g/mL), is included. Alcohol intake (g/kg) was calculated by the following equation: ((alcohol fluid intake in mL) × (0.2 × 0.789))/(bodyweight in kg). Preference for alcohol (%) was calculated according to the following equation: (alcohol intake in mL)/((alcohol intake in mL) + (water intake in mL)) × 100. Alcohol intake was calculated per rat per session and was averaged per week. To select the rats that consistently consumed low or high levels of alcohol throughout the experiment, the rats within each batch were ranked from low to high based on the rats' average alcohol intake per week over the first 4 weeks of alcohol consumption and were assigned ranking scores accordingly. These weekly ranking scores were then summed to calculate a total rank score for each rat. This was in turn used to select the 25% lowest alcohol drinking rats (LD) and the 25% highest (HD) alcohol drinking rats, that is, based on a quartile split (Spoelder et al. 2017; Spoelder et al. 2015). Moreover, for LD average alcohol consumption levels across the first 4 weeks of drinking had to be ≤ 0.4 g/kg for batch 1 or ≤ 1.2 g/kg for batch 2 and average alcohol consumption during the first 4 weeks had to exceed 1.4 g/kg for batch 1 or 2.0 g/kg for batch 2 for rats to be considered high drinkers.

### 2.4 Surgeries

The animals were anesthetized with an intraperitoneal injection of a mixture of ketamine (75 mg/kg, Vetoquinol) and dexdomitor (0.25 mg/kg, Orion Pharma) and placed in a stereotaxic apparatus (David Kopf Instruments, United States). An incision was made along the midline of the skull, and craniotomies were made bilaterally above the brain region of interest. The anatomical projection from the sensorimotor cortex to the DLS was confirmed by local infusion of 2.0 μL of a mixture of GFP‐tagged retrograde travelling rabies virus (Rabies‐SadDG‐GFP; 2.0 × 10^8^ genomic copies/mL) and AAV‐hSyn‐mCherry (3.4 × 10^12^ genomic copies/mL) at a rate of 0.2 μL/min into the DLS using the coordinates: AP –0.4, ML ± 3.5, DV –5.1 from skull, relative to Bregma as verified using ink injections. For electrophysiology experiments, after the first month of alcohol consumption, 10 selected LD, 18 selected HD and the 20 water drinking control rats received double bilateral intracranial injections of an adeno‐associated viral vector driving the expression of light‐activatable cation channel Channelrhodopsin (AAV‐5 CamKII‐hChR2‐eYFP; 8.5 × 10^12^ genomic copies/mL; lot AV4316). The infusions (2 × 0.4 μL at a rate of 0.2 μL/min) were targeted at the somatosensory cortex, using the following coordinates: AP +2.8, ML + 2.7, DV –2.9 from skull, relative to Bregma and AP +2.0, ML + 3.2, DV –2.4 from skull, relative to Bregma. The injectors were withdrawn 10 min post‐infusion. The rats were allowed to recover for at least 1 week before behavioral testing continued. We used the Paxinos and Watson (2004) to determine the stereotaxic coordinates.

### 2.5 Brain‐Slice Electrophysiology

#### 2.5.1 Slicing Procedures

Three to five days after the last alcohol consumption session, the rats were anesthetized with an intraperitoneal injection of sodium pentobarbital (Euthasol 20%, 0.1 mL, i.p.) and transcardially perfused with ice‐cold carbogenated (95% O_2_, 5% CO_2_) slicing solution containing (in mM): choline chloride 92; ascorbic acid 10; CaCl_2_ 0.5; glucose 25; HEPES 20; KCl 2.5; *N*‐acetyl L cysteine 3.1; NaHCO_3_ 25; NaH_2_PO_4_ 1.2; NMDG 29; MgCl_2_ 7; sodium pyruvate 3; thiourea 2. Dissected brains were submerged in ice‐cold carbogenated slicing solution and 250‐μm‐thick coronal slices of the dorsolateral striatum were cut using a vibratome (1200 VTs, Leica, Rijswijk, the Netherlands). Slices recovered for 30 min at 36°C in carbogenated solution of identical composition. Thereafter, slices were maintained at room temperature in carbogenated incubation solution containing (in mM): ascorbic acid 3; CaCl_2_ 2; glucose 25; HEPES 20; KCl 2.5; NaCl 92; NaHCO_3_ 20; NaH_2_PO_4_ 1.2; NMDG 29; MgCl_2_ 2; sodium pyruvate 3; and Thiourea 2.

#### 2.5.2 Recordings

During recordings, slices were immersed in artificial cerebrospinal fluid (ACSF) containing (in mM): CaCl2 2.5; glucose 11; HEPES 5; KCl 2.5; NaCl 124; NaHCO3 26; NaH2PO4 1; MgCl2 1.3 and were continuously superfused at a flow rate of 2.5 mL min^–1^ at 28°C–30°C. Bicuculline (20 μM; Tocris Bioscience) was bath‐infused to block GABA_A_ receptor‐mediated inhibitory postsynaptic currents. Medium spiny neurons in the dorsolateral striatum were visualized by video microscopy (×40 lens; THC‐200 Olympus microscope, France) with infrared differential interference contrast (DIC). Patch‐clamp recordings were made using glass borosilicate glass pipettes (3–5MΩ) filled with an internal medium containing (in mM) Cs‐methanesulfonate 139, CsCl 5, HEPES 10, EGTA 0.2, creatine phosphate 10; Na_2_ATP 4; Na_3_GTP 0.3; MgCl_2_ 2 (pH 7.2–7.4, Osmolarity 300–310 mOsm). Whole‐cell recordings were collected using an Axoclamp 2B amplifier (Molecular Devices). Data were stored and analyzed using pClamp software (Molecular Devices). The signal was amplified, filtered at 2.9 kHz and digitized at 20 kHz. Experimental recordings were initiated after 10 min. Series resistance was monitored online with a hyperpolarizing –4‐mV step pulse. Recordings that showed more than 20% variation in series resistance were excluded from the analysis. Light pulses (1–10 ms, 470 nM) were delivered with a LED illumination system (CoolLED, UK) to evoke optical‐induced post synaptic currents. CNQX (10 μM, Tocris Bioscience) was used to identify the nature of these optical‐induced post synaptic currents.

#### 2.5.3 Paired Pulse Protocol

Recordings were made in voltage clamp at –70 mV. Two brief light pulses (1–10 ms, 470 nM) with a fixed inter stimulation interval of 100 ms were delivered to measure paired‐pulse responses. Paired‐pulse ratios (PPR) were calculated as the ratio between the second and first excitatory postsynaptic currents (average of 10 sweeps).

#### 2.5.4 AMPA/NMDA Protocol

Recordings for AMPA and AMPA+NMDA mediated currents were made at –65 and +40 mV, respectively. To isolate the NMDA component, peak amplitudes of AMPA currents at –65 mV were compared to AMPA+NMDA currents at a 100‐ms timepoint post stimulus onset.

### 2.6 Data Analysis and Statistics

All data was normally distributed, except for the amplitude data (first experiment, Figure 2). Therefore, the nonparametric Mann–Whitney test was performed to determine the effect of CNQX. The alcohol intake and preference data were analyzed by repeated measures ANOVA with time (in weeks) as the within‐subjects factor and group (low or high drinkers) as the between‐subjects factor. The different parameters derived from the electrophysiological recordings were all analyzed using one‐way ANOVA's with group (alcohol drinkers or water controls) and/or subgroup (low drinkers, high drinkers or water controls) as the between‐subjects factor.

Mauchly's test of sphericity was used to determine if variances of the differences between timepoints were equal. Whenever the assumption of sphericity was violated, a Greenhouse–Geisser correction was used. Corrected degrees of freedom are presented, rounded to the nearest integer.

The data are expressed as mean ± SEM, unless otherwise stated. Data were visualized using Graphpad Prism (Version 8.3.0, Graphpad Software Inc., USA) and statistical analyses were performed using SPSS for Windows (25.0.0.1, IBM Corp., USA). Significance was accepted at *p* < 0.05.

## 3 Results

### 3.1 Verification of Sensorimotor Input to the DLS

Histological analysis for the brain sections of rats that received an intra‐DLS infusion of the GFP‐tagged retrograde labelling rabies virus confirmed innervation of the DLS by cortical areas, in particular the sensorimotor cortex (Figure 2A). Recordings from DLS neurons upon stimulation with 470‐nm light in rats that received intracerebral infusions of CaMKII‐ChR2‐eYFP expressing AAV into the sensorimotor cortex revealed light‐evoked excitatory postsynaptic currents (EPSCs; Figure 2C,D). Similar recordings 10 min after wash‐in of CNQX (10 μM) showed that light‐stimulation evoked EPSCs were blocked (*U* = 360, *p* < 0.001; Figure 2C,D), indicating that the corticostriatal projections were glutamatergic.

### 3.2 Alcohol Consumption

**FIGURE 3:**
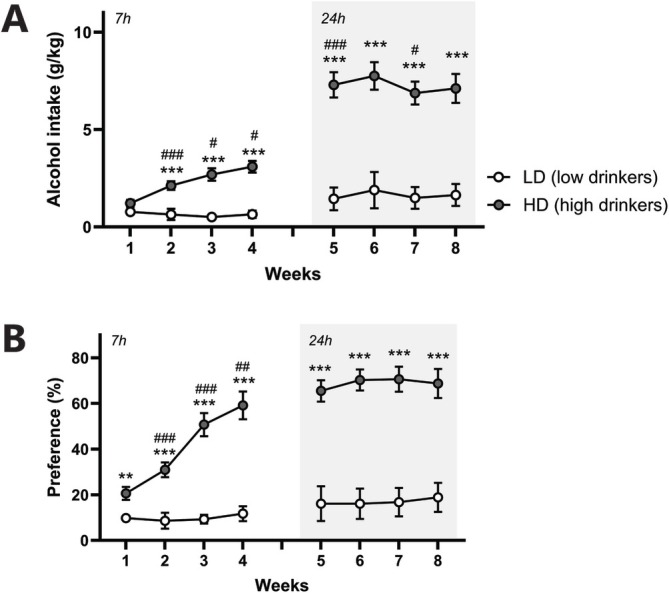
Alcohol consumption. Average alcohol intake (g/kg, A) and alcohol preference (% of total volume consumed, B) across the 8 weeks of voluntary home cage drinking for the low (*n* = 10) and high (*n* = 18) alcohol drinking subgroups. The sessions were 7 h in duration for the first 4 weeks and were thereafter extended to 24 h for weeks five to eight. Data are expressed as mean ± SEM. ** or ***denotes significance between low and high alcohol drinking rats at a *p* < 0.01 or *p* < 0.001 level, respectively. #, ##, or ### denotes significance from the preceding week for the high alcohol drinkers at a *p* < 0.05, *p* < 0.01, or *p* < 0.001 level, respectively.

The average weekly levels of alcohol intake and the preferences for alcohol were calculated for the selected low and high alcohol drinking rats (see Figure 3A,B). For both alcohol intake (g/kg) and alcohol preference (%) statistical analyses revealed significant overall effects of time (*F*_time intake_ (3,71) = 60.5, *p* < 0.001; *F*_time preference_ (4101) = 32.8, *p* < 0.001), significant overall group effects (*F*_group intake_ (1,25) = 97.6, *p* < 0.001; *F*_group preference_ (1,25) = 133.5, *p* < 0.001) and significant group × time interactions (*F*_time × group intake_ (3,71) = 29.4, *p* < 0.001; *F*_time × group preference_ (4101) = 16.9, *p* < 0.001), emphasizing the difference between the selected groups. The difference between low and high alcohol drinking rats was already clear and consistent after 4 weeks of alcohol consumption (HD consumed 475% more alcohol than LD in week 4), at the time when the intracranial infusions of the AAV particles were performed. In contrast to the low alcohol drinking rats, the high drinkers increased their alcohol intake and preference over time and, in line with previous findings (Spoelder et al. 2017; Spoelder et al. 2015), particularly so at the transition from 7 h to 24 sessions.

### 3.3 Weakened Excitatory Sensorimotor Inputs to DLS Neurons After Alcohol Consumption

Analysis of the basic electrophysiological properties for the neurons we recorded, revealed no difference between the rats that consumed alcohol and the water control animals in capacitance (*F*_group_ (1,27) = 0.32, N.S.), *R*_membrane_ (*F*_group_ (1,24) = 1.28, N.S.), or Leak current (*I*_leak_; *F*_group_ (1,33) = 1.57, N.S.) (Figure S1). These findings suggest that alcohol consumption does not affect the passive properties of medium spiny neurons in the DLS.

**FIGURE 4:**
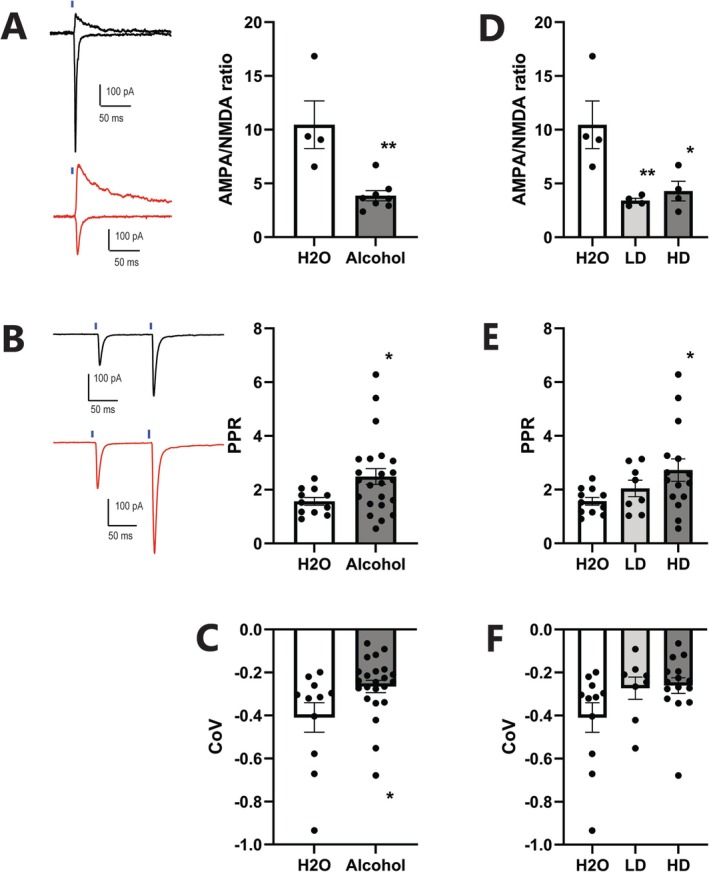
Electrophysiology data. A–C represent the average data for the water control group and the alcohol consuming rats, irrespective of their level of alcohol consumption. (D–F) the data for the low and high alcohol consuming rats are separately represented. Shown are in A and D the AMPA/NMDA ratio data, with representative traces on the left, in B and E, the paired pulse ratio (PPR) data, with representative traces on the left, and in C and F, the coefficient of variance (CoV) data. The black traces represent the water control rats and the red traces represent the alcohol consuming rats, respectively. The blue rectangles in the traces indicate the stimulation moments. Number of cells/animals/batches: PPR/CoV water—11 cells, 8 rats, 2 batches; PPR/CoV alcohol—23 cells, 12 rats, 2 batches; A/N ratio water—5 cells, 5 rats, 2 batches; A/N ratio alcohol—8 cells, 5 rats, 2 batches. Data are expressed as mean ± SEM. * or ** denotes significance between alcohol consuming rats and water controls at a *p* < 0.05 or *p* < 0.01 level, respectively.

Prolonged voluntary alcohol consumption did not affect the amplitude of single, light‐evoked EPSCs in medium spiny neurons in the DLS (162.9 ± 28.1 vs. 133.2 ± 20.2 pA for control vs. EtOH respectively; *F*_group_ (1,33) = 0.72, N.S.). However, alcohol consumption reduced the AMPA/NMDA ratio by 63% (*F*_group_ (1,11) = 16.4, *p* < 0.01; Figure 4A), indicative of LTD, potentially involving a postsynaptic mechanism. In addition, alcohol exposure increased the paired pulse ratio by 59% (*F*_group_ (1,33) = 4.38, *p* < 0.05; Figure 4B) and reduced the coefficient of variance by 35% (*F*_group_ (1,33) = 5.24, *p* < 0.05; Figure 4C), indicative of presynaptic effects of alcohol consumption.

When we took the division into low and high alcohol drinking rats into account in the analysis of the synaptic properties of sensorimotor projections to the DLS (Figure 4D–F), there was a significant effect of subgroup on the AMPA/NMDA ratio (*F*_subgroup_ (2,11) = 7.65, *p* < 0.05; Figure 4D). Post hoc pairwise comparisons showed a reduction in the AMPA/NMDA ratio for both the low and high alcohol drinking rats, by 67% and 59%, respectively, relative to the water controls. Furthermore, there was a trend toward a significant effect of subgroup on the paired pulse ratio (*F*_subgroup_ (2,32) = 3.10, *p* = 0.059; Figure 4E) and post hoc pairwise comparisons showed that the increase in the paired pulse ratio was particularly pronounced in the DLS of rats that consumed the highest level of alcohol (by 74%), when compared to the animals in the water control group (see Figure 4E). By contrast, there was no significant difference between the subgroups for the coefficient of variance data (*F*_subgroup_ (2,33) = 2.55, *p* = 0.09; Figure 4F).

## 4 Discussion

In the current study we determined the impact of prolonged alcohol consumption on synaptic properties in the projections from the sensorimotor cortex to the DLS in rats. Prolonged voluntary consumption of alcohol in rats reduced AMPA/NMDA ratios in the DLS, indicative of LTD. Contrary to our hypothesis, these effects were, however, not dependent on the level of alcohol consumption as the reduction in AMPA/NMDA ratios was comparable for the low and high alcohol drinking rats. Moreover, the reduced probability of presynaptic glutamate release, as evident from an increase in paired‐pulse facilitation, which was particularly pronounced for high alcohol drinking rats, and the reduced coefficient of variance suggest that voluntary alcohol consumption also caused presynaptic changes (Linders et al. 2022). Taken together, these findings underline the impact of alcohol consumption on corticostriatal plasticity.

The present results demonstrate synaptic plasticity in optogenetically identified cortico‐striatal pathways after prolonged alcohol consumption. The observed reduction in AMPA/NMDA ratio, often reflective of a postsynaptic receptor mechanism (Linders et al. 2022), was accompanied by a reduction of presynaptic glutamate release. Reduced glutamatergic control of the sensorimotor cortex to the DLS may contribute to the shift towards compulsive and/or habitual alcohol seeking (as discussed later), but these effects were largely independent of the individual levels of alcohol consumption. Although our experiments do not directly address the relation between synaptic plasticity and addiction‐like behaviors, there is ample evidence to show that addiction‐like behaviors including habitual alcohol seeking and/or loss of control over alcohol seeking that emerge upon prolonged exposure to alcohol (Augier et al. 2018; Corbit et al. 2012; Giuliano et al. 2021; Hopf et al. 2010; Lesscher et al. 2010; Spoelder et al. 2017; Spoelder et al. 2015) rely on the DLS (Corbit et al. 2012; Patton et al. 2021). Moreover, habit formation was previously shown to emerge upon voluntary alcohol consumption but comparably so for low and high alcohol drinking rats (Smeets et al. 2022).

The reduction in strength of sensorimotor cortex inputs onto DLS medium spiny neurons upon chronic alcohol consumption is in apparent contrast to previous studies that reported impaired LTD in the dorsal striatum after prolonged alcohol exposure (Cui et al. 2011; DePoy et al. 2013; DePoy et al. 2015; Xia et al. 2006). Our results may reflect the impact of both alcohol exposure and the acute withdrawal from alcohol exposure, because our recordings were made within 3–4 days after the last alcohol consumption session. The differential effects of alcohol exposure on synaptic plasticity in the dorsal striatum between our studies and findings described previously (Cui et al. 2011; DePoy et al. 2013; DePoy et al. 2015; Xia et al. 2006) may be related to the method of exposure, i.e., voluntary consumption or forced exposure through drinking water or ethanol vapor. Indeed, neuroadaptations in response to alcohol are known to differ for voluntary or forced ethanol exposure (de Guglielmo et al. 2022).

The dorsolateral striatum integrates multisensory information with motor commands to strengthen and fine‐tune actions that lead to the acquisition of rewards (Choi et al. 2017; Graybiel 2008; Haber 2016). A shift from ventral to dorsal striatal control has been posited to mediate the transition from voluntary substance use to loss of control over and habitual substance use (Everitt 2014; Everitt et al. 2008; Everitt and Robbins 2005, 2015). There is compelling evidence that the changes in synaptic plasticity in the dorsal striatum as a consequence of extended exposure to alcohol are paralleled by the development of habitual and compulsive substance seeking. However, the exact mechanisms through which synaptic plasticity changes in the dorsal striatum, such as described here, contribute to the development of addictive behavior remain largely unknown. Work by Corbit et al. has for instance shown that extended alcohol consumption results in habitual ethanol seeking, which was dependent on the dorsal striatum (Corbit et al. 2012, 2014). In line with these findings, a recent study implicated BDNF in prefrontal cortical projects toward the DLS in habitual alcohol seeking (Gunasekaran et al. 2025). Moreover, multiple studies have shown that the dorsal striatum regulates binge drinking and (compulsive) alcohol consumption (Bahi and Dreyer 2013; Bauer et al. 2021; Darcq et al. 2016; Giuliano et al. 2019; Jeanblanc et al. 2015; Patton et al. 2021). Although we did not determine addictive behavior in the rats in the current study, previous studies from our lab have shown that voluntary home cage alcohol consumption in a limited access choice paradigm results in loss of control over alcohol seeking, using both quinine adulteration and operant conditioned suppression task (Spoelder et al. 2017; Spoelder et al. 2015). Moreover, a recent study revealed increased brain iron accumulation in the dorsal striatum in individuals with AUD, which was related to compulsive drinking behavior (Tan et al. 2023).

## 5 Conclusions

Taken together, these findings provide further evidence for the important role of the DLS in addictive behavior. Specifically, the current results point toward a breakdown of sensorimotor drive toward the DLS, which might contribute to the development of maladaptive alcohol drinking patterns. Future in vivo functional anatomical studies are required to better understand how synaptic plasticity changes in the DLS contribute to alcohol addiction.

## Author Contributions

**Johanna A. S. Smeets:** conceptualization, investigation, visualization, writing – original draft. **Geert M. J. Ramakers:** funding acquisition, conceptualization, supervision, methodology, formal analysis, writing – original draft, writing – review and editing. **Frank J. Meye:** supervision, methodology, writing – review and editing. **Anne‐Marie Baars:** investigation. **Roger A. H. Adan:** funding acquisition, conceptualization, supervision, writing – review and editing. **Louk J. M. J. Vanderschuren:** funding acquisition, project management, conceptualization, supervision, writing – review and editing. **Heidi M. B. Lesscher:** funding acquisition, project management, conceptualization, supervision, formal analysis, visualization, writing – original draft, writing – review and editing.

## Conflicts of Interest

The authors declare no conflicts of interest.

## Funding

This work was supported by ZonMw (912.14.093).

## Supporting information


**Figure S1:** Average capacitance (A), *R*_membrane_ (B), and *I*_leak_ (C) for water control rats and rats that consumed alcohol. Number animals/batches/cells: water—4 rats, 2 batches, 5 cells (capacitance and *R*_membrane_) and 11 cells (*I*_leak_); alcohol—12 rats, 2 batches, 23 cells (capacitance and *I*_leak_) and 20 cells (*R*_membrane_). Data are expressed as mean ± SEM

## Data Availability

The data that support the findings of this study are available online, through the following link: https://doi.org/10.34894/KHFKF7.
