# Supplementary figures and images for: Impact of Voluntary Alcohol Consumption on Corticostriatal Plasticity in Rats
Source: Eur J Neurosci. 2026 Jul 2;64(1):e70602. doi: 10.1111/ejn.70602 (PMC13324971; doi:10.1111/ejn.70602)

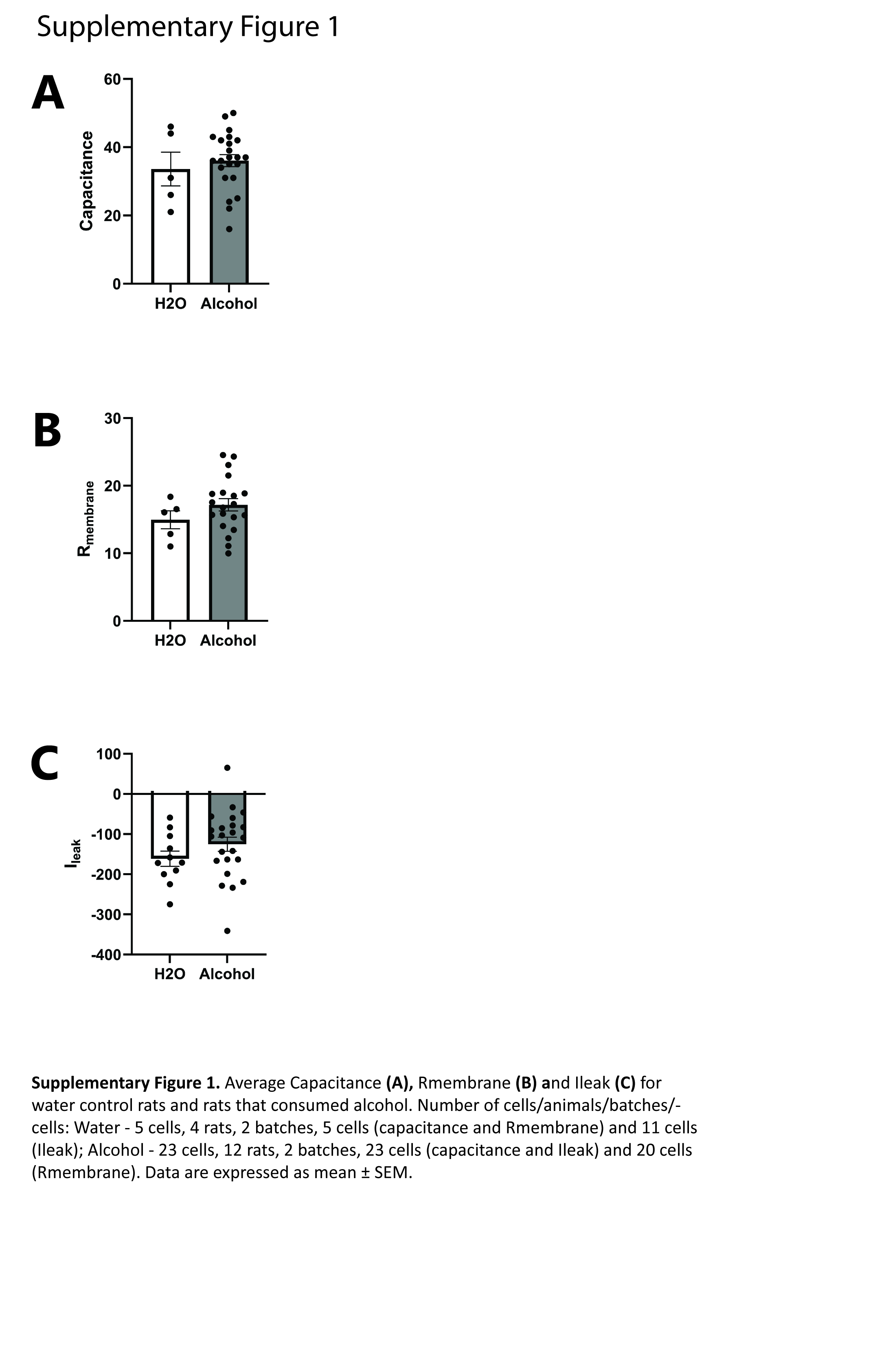

Supplement: Supplementary file 1 — Figure S1: Average capacitance (A), R membrane (B), and I leak (C) for water control rats and rats that consumed alcohol. Number animals/batches/cells: water—4 rats, 2 batches, 5 cells (capacitance and R membrane) and 11 cells (I leak); alcohol—12 rats, 2 batches, 23 cells (capacitance and I leak) and 20 cells (R membrane). Data are expressed as mean ± SEM [file EJN-64-0-s001.tif]
